# Sinonasal Inverted Papillomas: Predictors of Recurrence and Malignant Transformation

**DOI:** 10.3390/life16030442

**Published:** 2026-03-09

**Authors:** Ionut Tanase, Mircea-Sorin Ciolofan, Codrut-Caius Sarafoleanu, Carmen Aurelia Mogoantă, Florentina-Carmen Badea, Constantin-Ioan Busuioc, Shirley Tarabichi, Alex Milea, Ilona Mihaela Liliac, Dan Iovanescu, Gheorghe Iovanescu, Gabriela-Cornelia Musat

**Affiliations:** 1 Department of Otorhinolaryngology, “Carol Davila” University of Medicine and Pharmacy, 050474 Bucharest, Romania; ionut.tanase@umfcd.ro (I.T.); codrut.sarafoleanu@umfcd.ro (C.-C.S.); gabriela.musat@umfcd.ro (G.-C.M.); 2 Department of Otorhinolaryngology, University of Medicine and Pharmacy of Craiova, 200349 Craiova, Romania; sorin.ciolofan@umfcv.ro; 3 ENT Department, Medicover Hospital, 020331 Bucharest, Romania; 4 Department of Pathology, Sfanta Maria Hospital, 011172 Bucharest, Romania; busuioc.constantin@gmail.com; 5 Doctoral School, Faculty of Medicine, “Carol Davila” University of Medicine and Pharmacy, 050474 Bucharest, Romania; shirley.tarabichi@gmail.com (S.T.); mileaalex1993@gmail.com (A.M.); 6 Department of Histology, Faculty of Medicine, University of Medicine and Pharmacy of Craiova, 200349 Craiova, Romania; ilona.liliac@umfcv.ro; 7 Department of Otorhinolaryngology, “Victor Babes” University of Medicine and Pharmacy, 300113 Timisoara, Romania; dan.iovanescu@umft.ro (D.I.); giovanescu@umft.ro (G.I.)

**Keywords:** inverted papilloma, recurrence, malignant transformation, p16, HPV

## Abstract

Sinonasal inverted papillomas (IPs) are rare benign tumors with ~15% postoperative recurrence and a ~8% risk of malignant transformation, with human papillomavirus (HPV) reported as a risk factor in IP malignant transformation. To evaluate clinical, molecular and immunohistochemical factors associated with recurrence and malignant transformation in IPs. We retrospectively analyzed 73 patients with histologically confirmed IPs that were treated at three tertiary ENT centers, including radiologic data, HPV DNA detection and p16 immunohistochemistry. Univariate analysis was used to identify factors associated with recurrence and malignant transformation, and restricted exploratory multivariable logistic regression models were used to assess recurrence while minimizing overfitting. Fourteen recurrences (19%) were associated with longer symptom duration (*p* = 0.003), smoking (*p* = 0.03), advanced Krouse stage (III–IV) (*p* < 0.001), frontal sinus origin (*p* = 0.02), HPV+ DNA (50% vs. 22%, *p* = 0.048), and p16 loss/reduced expression (*p* = 0.006). Nine recurrences transformed into carcinoma (12%) and were associated with smoking (*p* = 0.01). HPV+ was not associated with malignancy (*p* = 1.00). Recurrence was associated with the advanced stage of the IP, tobacco use, longer symptom duration, frontal sinus origin, HPV+, and p16 loss/reduced expression.

## 1. Introduction

IPs are epithelial tumors that are histologically benign in most cases, yet they clinically behave as locally aggressive lesions with a well-recognized risk of postoperative recurrence and malignant transformation. They arise from the Schneiderian mucosa of the nasal cavity and paranasal sinuses and occur more commonly in middle-aged men (~3–4:1 M:F); there is a ~15% chance of recurrence, and ~8% become carcinoma [1]. IPs are endoscopically resected, with identification and removal of the attachment site. A long-term surveillance follows. Predicting which patients are more likely to recur or become carcinogenic is challenging, as imaging does not fully capture IP biological heterogeneity [2]. While HPV has been implicated in some cases—particularly those with dysplasia or carcinoma—its prevalence and relevance vary widely among studies, and its association with recurrence or malignant transformation is inconsistent [3,4,5]. p16 (often used as an HPV-related marker in other head and neck sites) inconsistently correlates with HPV status in IPs and may reflect broader cell-cycle pathway changes [6,7]. We add 73 additional cases of sinonasal IP papilloma to the literature, noting clinical, radiologic, and pathological factors such as HPV and p16 with respect to recurrence and malignant transformation.

## 2. Methods

### 2.1. Study Design

This retrospective study was conducted between 1 January 2015 and 31 August 2025 and included cases from three tertiary referral centers from Romania: the Department of Otorhinolaryngology, “Sfanta Maria” Clinical Hospital, Bucharest; the Department of Otorhinolaryngology, County Emergency Clinical Hospital, Craiova; and the Department of Otorhinolaryngology of the Emergency Municipal Hospital, Timisoara. All patients underwent endoscopic evaluation, imaging, histopathological assessment, and surgical treatment in the participating centers, according to local clinical practice.

The study received approval from the Institutional Ethics Committees of all three participating centers: approval no. 20326/29.07.2024 for “Sfanta Maria” Clinical Hospital; approval no. 3976/25.01.2024 for the County Emergency Clinical Hospital, Craiova; and approval no. 7/14.01.2026 for the Emergency Municipal Hospital, Timisoara.

### 2.2. Patient Selection

A total of 181 patients with a histopathologic diagnosis of sinonasal inverted papilloma were identified in the institutional databases during the study period. After applying the eligibility criteria below, 73 patients were included in the final study cohort: 28 from “Sfanta Maria” Clinical Hospital, Bucharest; 26 from the Emergency Municipal Hospital, Timisoara; and 19 from the County Emergency Clinical Hospital, Craiova.

The eligibility criteria were as follows:

Inclusion criteria:

- Age ≥ 18 years;
- Primary (newly diagnosed) sinonasal inverted papilloma;
- Surgical treatment performed in one of the participating centers;
- No history of prior sinonasal surgery for inverted papilloma;
- Adequate clinicoradiological documentation (preoperative endoscopy, computed tomography—CT; magnetic resonance imaging—and MRI when available/indicated);
- Available HPV DNA testing results;
- Evaluable p16 immunohistochemistry in archival specimens;
- Documented postoperative follow-up allowing assessment of recurrence and/or malignant transformation.

Exclusion criteria:

- Revision/recurrent cases previously treated in other institutions and referred for further management;
- Non-surgical management;
- Insufficient clinical/imaging documentation to confirm eligibility and staging;
- Missing HPV results;
- Non-evaluable archival material for p16 immunohistochemistry (e.g., unavailable block/slide or inadequate tissue preservation);
- Insufficiently documented follow-up to reliably ascertain recurrence and/or malignant transformation.

### 2.3. Data Collection

The following parameters were extracted from medical records and imaging archives:

- Demographic data (age and sex);
- Clinical presentation and laterality;
- Endoscopic findings;
- Imaging features on CT and MRI (tumor extension, hyperostosis, and bone remodeling);
- Site of origin;
- Krouse staging [8];
- Treatment modality;
- Postoperative follow-up duration;
- Recurrence and malignant transformation.

As this was a retrospective study, patients did not follow a predefined or standardized follow-up protocol. Instead, postoperative monitoring reflected the routine clinical practice of the three participating centers. Based on the medical records, most patients underwent an early postoperative nasal endoscopy at approximately 1 month, followed by evaluations every 3 months during the first year, every 6 months during the next two years, and annually thereafter in clinically stable cases. Additional visits were scheduled when patients developed new or recurrent symptoms. Imaging studies (CT or MRI) were performed when endoscopic findings or clinical evolution raised suspicion for residual or recurrent disease.

### 2.4. HPV Detection and Genotyping

HPV DNA detection was performed on archival formalin-fixed, paraffin-embedded (FFPE) tumor samples from all 73 patients. All molecular analyses were performed retrospectively on stored FFPE blocks. DNA was extracted using a standard commercial kit and subjected to polymerase chain reaction (PCR) amplification that targeted the L1 region. Positive samples were then genotyped, identifying both high- and low-risk HPV types using the geneMAP^TM^ HPV 29 Genotyping Kit (Genmark, Instanbul, Turkey). The high-risk group included *HPV16*, *HPV18*, *HPV26*, *HPV31*, *HPV33*, *HPV35*, *HPV39*, *HPV45*, *HPV51*, *HPV52*, *HPV53*, *HPV56*, *HPV58*, *HPV59*, *HPV66*, *HPV68*, and *HPV73*. The low-risk group included: *HPV6/11*, *HPV40*, *HPV42*, *HPV43*, *HPV44*, *HPV54*, *HPV61*, *HPV69*, *HPV70*, and *HPV81/82*.

### 2.5. Immunohistochemistry (IHC)

Here, 4–5 μm-thick sections from FFPE blocks were deparaffinized, rehydrated, and subjected to heat-induced epitope retrieval in a citrate buffer (pH 6.0). IHC was performed using a monoclonal mouse anti-human p16INK4a antibody clone RM267 (producer: BioSB, Santa Barbara, CA, USA).

Scoring: p16 expression was scored as retained (diffuse strong nuclear and cytoplasmic staining in ≥70% of tumor cells) or loss/reduced (absent or focal weak staining in <70% of tumor cells), following established criteria for sinonasal tumors [9].

For cases exhibiting malignant transformation, a paired assessment of benign and malignant components was performed to evaluate biomarker shifts.

### 2.6. Outcome Measures

Primary outcomes:

- Recurrence was defined as endoscopically and/or radiologically suspected tumor regrowth that was confirmed histologically after the index surgery. Given the retrospective design, events detected within the first postoperative year may represent persistent/residual disease rather than true de novo recurrence. However, for consistency with prior series, all histologically confirmed postoperative regrowth events were analyzed as recurrence.
- Malignant transformation, defined as synchronous carcinoma or carcinoma during follow-up.

Secondary outcomes: association of recurrence with HPV status and p16 expression.

### 2.7. Statistical Analysis

Statistical analysis was performed using IBM SPSS Statistics version 26.0. Descriptive statistics were expressed as means ± SD or median (IQR), as appropriate. Continuous variables (e.g., age, symptom duration, and time to recurrence) were compared using independent samples *t*-tests or Mann–Whitney U tests based on the results of the Shapiro–Wilk normality test. Categorical variables (e.g., recurrence, HPV status, and p16 expression) were analyzed using Chi-square or Fisher’s exact test, as appropriate. Associations between biomarker status and clinical outcomes were evaluated using Chi-square or Fisher’s exact test, as appropriate. Univariate analyses were used to explore factors associated with recurrence and malignant transformation. In addition to univariate analyses, we performed two restricted exploratory multivariable logistic regression models for recurrence. Because the number of recurrent events was limited, each model was intentionally restricted to two clinically relevant covariates to minimize overfitting: Model A included advanced Krouse stage (III–IV vs. I–II) and HPV DNA positivity (positive vs. negative), while Model B included advanced Krouse stage (III–IV vs. I–II) and p16 expression status (loss/reduced vs. retained). Adjusted odds ratios (ORs) with 95% confidence intervals (CIs) were calculated. No multivariable model was performed for malignant transformation due to the limited number of malignant events. A *p*-value < 0.05 was considered statistically significant.

## 3. Results

### 3.1. Patient Demographic and Clinical Presentation

A total of 73 patients with histologically confirmed IP were included in the final analysis. The primary site of the IP was the maxillary sinus (in 36; 49%), ethmoid (in 18; 25%), lateral nasal wall (in 13, 18%), frontal sinus in four patients, and sphenoid sinus in two patients. Krouse stage: T1—14 (19%), T2—25 (34%), T3—23 (32%), and T4—11 (15%). Most of the patients were men (55 of 73; 75%); the mean age at diagnosis was 44.6 ± 13.3 years. The duration of the symptoms ranged from 3 months to 7 years, with a median of 18 months (IQR 6–36). The disease was predominantly unilateral (left 52%, one patient had bilateral disease). Symptoms were nasal obstruction (in 68; 93%), rhinorrhea (in 50, 69%), headache (in 20, 27%), and epistaxis (in 12; 16%). Fourteen patients reported hypoacusis, and 7 reported anosmia (10%). Five patients (7%) were asymptomatic at diagnosis and were identified incidentally during evaluation for unrelated complaints or on imaging/endoscopic examination. Smoking was reported in 42 (58%). Allergic rhinitis was undetermined in 24 (33%), positive in 22 (30%), and negative in 27 (37%).

**Figure 1:**
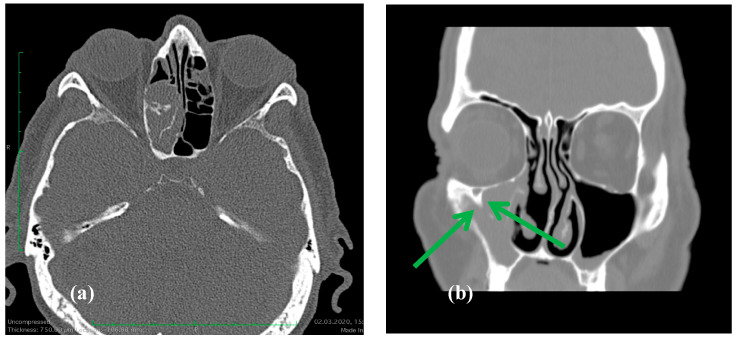
(**a**). Axial CT showing a right ethmoid sinus IP with focal hyperostosis along the lateral ethmoid wall, indicative of the tumor’s likely attachment site, a characteristic imaging feature of IP. (**b**). Coronal CT showing an inverted papilloma arising from the right maxillary sinus. Note the focal area of hyperostosis near the infraorbital canal (green arrows). The need to preserve the infraorbital nerve makes complete IP removal more difficult, increasing the risk of residual disease and recurrence.

Imaging: All patients underwent CT, which showed focal hyperostosis that was consistent with the suspected site of attachment (Figure 1). MRI was obtained selectively when orbital or skull base extension was suspected.

### 3.2. Resection and Outcomes

Endoscopic resection was usually the primary treatment, adding an external approach in four cases with extensive tumor involvement or difficult access sites. Operative reports indicated gross total resection in all cases, without major complications. The mean follow-up duration was 61.2 months, and the median follow-up was 51 months (IQR 24–84), ranging from 6 to 128 months. The recurrence rate in this inverted papilloma cohort was 19% (14/73). Most recurrences were managed with revision endoscopic surgery; an external approach was added in 3 of 14 recurrent cases (21%), reflecting greater complexity and extent of disease. The time to recurrence ranged from 8 months to 4 years (mean 2.1 years).

**Figure 2:**
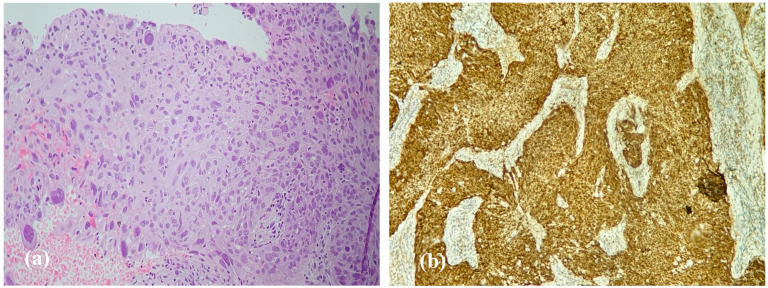
Histopathology and p16 immunohistochemistry in inverted papilloma with malignant transformation (**a**). H&E, ×40. Inverted papilloma with areas of squamous cell carcinoma; atypical pleomorphic nuclei are present. (**b**). p16 immunohistochemistry, ×20. Diffuse (“block-type”) nuclear and cytoplasmic staining in tumor cells.

Squamous cell carcinoma (SCC) consistent with malignant transformation was seen in 3 patients with IP at their first surgery, and occurred later in 6 other patients, at an interval of 1–5 years after initial resection (Figure 2a). SCCs were resected, and patients received either irradiation or chemoradiation.

### 3.3. Univariate Analysis of Factors Associated with Recurrence and Malignant Transformation

Recurrence was significantly associated with longer symptom duration (median 30 vs. 12 months, *p* = 0.003), smoking (86% vs. 51%, *p* = 0.03), advanced Krouse stage (III–IV: 86% vs. 34%, *p* < 0.001), and frontal sinus origin (21% vs. 2%, *p* = 0.02) (Table 1). Among the three recurrent cases with frontal sinus involvement, one patient required an external approach because the lesion extended into the lateral recess of the frontal sinus, beyond the limits of safe endoscopic access, while the other two were managed endoscopically with Draf IIB and Draf III procedures, respectively.

HPV+ DNA was more frequent among recurrent cases (50% vs. 22%, *p* = 0.048). Age and sex were not significantly associated with recurrence. Malignant transformation occurred in nine patients and was significantly associated with smoking (100% vs. 52%, *p* = 0.01). No statistically significant associations were seen between malignant transformation and age/sex, symptom duration, allergic rhinitis, laterality, Krouse stage, frontal sinus origin, combined surgical approach, or HPV+ DNA (Table 1).

### 3.4. Multivariable Logistic Regression Models for Recurrence

To explore whether the biomarker associations persisted after a limited adjustment for disease extent, we performed two restricted exploratory multivariable models for recurrence (Table 2). In model A (advanced Krouse stage + HPV DNA positivity), both variables remained associated with recurrence: advanced Krouse stage (adjusted OR = 5.84, 95% CI 1.31–26.07, *p* = 0.021) and HPV DNA positivity (adjusted OR = 4.12, 95% CI 1.02–16.61, *p* = 0.047). In model B (advanced Krouse stage + p16 status), both variables remained associated with recurrence: advanced Krouse stage (adjusted OR = 6.10, 95% CI 1.55–24.05, *p* = 0.010) and p16 loss/reduced expression (adjusted OR = 4.35, 95% CI 1.06–17.88, *p* = 0.041). Given the limited number of recurrent events, these adjusted findings should be interpreted cautiously.

### 3.5. HPV DNA Status

HPV DNA was detected in 20/73 tumors (27%). HPV positivity was more frequent in recurrent cases compared with non-recurrent cases (50.0% vs. 22.0%, Fisher’s exact test, *p* = 0.048). In contrast, HPV DNA positivity was not associated with malignant transformation (22.2% vs. 28.1%, Fisher’s exact test, *p* = 1.00) (Table 3).

Among HPV-positive tumors, HPV11 and HPV16 were the most common genotypes, and co-infection was uncommon (Table 3). Given the limited number of malignant events, no statistically robust conclusion can be drawn regarding HPV as a predictor of malignant transformation. These findings suggest a possible association between HPV infections and recurrence risk in inverted papilloma, while malignant progression likely involves additional molecular pathways.

### 3.6. Immunohistochemical Findings—p16 Expression

p16 was evaluated as a cell-cycle regulatory marker; in inverted papilloma, it does not reliably indicate transcriptionally active HPV. Diffuse (retained) p16 expression was observed in 58 of 73 tumors (79%), whereas 15 tumors (21%) demonstrated reduced or absent staining (p16 loss/reduced) (Figure 2b). Recurrence occurred in 7 of 15 p16-loss tumors (47%), compared with 7 of 58 p16-retained tumors (12%), indicating a strong and statistically significant association between p16 loss and recurrence (*p* = 0.006, Fisher’s exact test). This supports p16 loss as a potential prognostic marker of more aggressive clinical behavior, independent of HPV DNA status. In contrast, most malignant transformations occurred in the tumors with retained p16 expression (7 of 9), suggesting that p16 loss/reduced staining is primarily linked to recurrence rather than malignant progression in this small cohort.

Among the nine cases that underwent malignant transformation, paired immunohistochemical assessment of benign and malignant components was feasible. In most cases, p16 expression was seen in both the benign and malignant areas, while a smaller subset showed a reduced expression in the malignant component.

## 4. Discussion

IP is a benign epithelial neoplasm with a ~15% rate of postoperative recurrence and an ~8% rate of malignant transformation. Unlike the 1271 cases discussed in a 2025 meta-analysis [10], in this paper, we retrospectively analyze our 73 patients for whom we have sufficient clinical and imaging records.

The predominance of middle-aged males is as expected [11,12]. IPs in our cohort usually occurred in the maxillary and ethmoid sinuses, as expected and in agreement with larger series. The frontal and sphenoid sinuses are locations where complete resection, including the site of attachment, may be challenging [13,14]. In line with prior reports, CT frequently demonstrated focal hyperostosis, a useful imaging feature suggesting the site of the tumor attachment and supporting planning to remove the implantation area completely. Resection of the hyperostotic area together with the tumor reduces the risk of recurrence, as also described in previous reports. A feature that may contribute to recurrence is a submucosal spread that may result in residual IP despite apparent macroscopic clearance, underscoring the importance of meticulous margin assessment and the resection of the attachment site [15,16,17]. The regrowth that was detected within the first postoperative year may represent persistent/residual disease rather than true de novo recurrence, particularly in anatomically constrained attachment sites. In such locations (e.g., the frontal sinus posterior table/lateral recess, the infraorbital canal region, or the sphenoid sinus in proximity to the skull base and the internal carotid artery), the extent of drilling is sometimes intentionally limited to preserve critical structures, which may increase the risk of microscopic residual disease and early postoperative regrowth.

The clinical presentation in our cohort was usually a unilateral nasal obstruction and rhinorrhea, in line with reviews describing these tumors as frequently asymptomatic early on and progressively symptomatic as they enlarge [18,19].

The recurrence rate in this cohort was 19% (14/73), within the reported recurrence range (~10–25%) [20]. The recurrent cases were presented with a significantly longer pre-diagnostic symptom duration than the non-recurrent tumors (median 30 vs. 12 months, *p* = 0.003), supporting the concept that diagnostic delay may permit a wider mucosal and submucosal spread, as suggested in previous series [16]. The time taken before recurrence (~2.1 years) was comparable to long-term studies: recurrence may occur beyond five years and, occasionally, up to 7–10 years [21], reinforcing the need for prolonged surveillance. Smoking was also more frequent among recurrent patients (86% vs. 51%, *p* = 0.03). Advanced Krouse stage (III–IV) in the recurrent group (86% vs. 34%, *p* < 0.001) was as expected and reported by others: larger tumors may be technically more difficult to fully resect, including the attachment site and adjacent submucosal spread. Frontal sinus origin was, as expected, associated with recurrence (21% vs. 2%, *p* = 0.02) as discussed earlier [22].

Malignant transformation occurred in nine patients (12%), within the range of reported rates [10], and was significantly associated with smoking. HPV prevalence has been widely investigated as a potential etiologic and prognostic factor in IP, with heterogeneous results. HPV prevalence varies across studies due to heterogeneity in detection techniques, geographic variation, and differences in case selection [23,24]. In our IP cohort, HPV+ DNA was significantly associated with recurrence (50% in recurrent vs. 22% in non-recurrent, *p* = 0.048). In contrast, HPV status was not significantly associated with malignant transformation. This is consistent with the broader literature, where HPV has been variably linked to recurrence but demonstrates inconsistent predictive values for malignant progression [25,26].

Unlike in oropharyngeal carcinomas [27,28], p16 in IP is not a simple surrogate marker of transcriptionally active HPV. Importantly, in IP, reduced/absent p16 staining may reflect HPV-independent cell-cycle pathway alterations rather than HPV-driven oncogenesis. One plausible explanation is *CDKN2A* (p16) inactivation (e.g., deletion or promoter methylation) with a downstream dysregulation of the *RB* checkpoint, which could contribute to a less stable epithelial phenotype and a higher propensity for postoperative regrowth [29]. Because molecular profiling for *CDKN2A/TP53/EGFR* or related pathway alterations was not available in the present study, this biological interpretation remains hypothesis-generating and warrants confirmation in prospective, molecularly characterized cohorts. The loss of p16 expression was associated with a markedly increased recurrence risk in our cohort (47% vs. 12%, *p* = 0.006). Menendez [30] similarly reported a strong link between reduced p16 staining and recurrence. Similar observations from other molecular pathology studies [31] exist. The p16 status did not show a clear association with malignant transformation, as most malignant cases retained p16 expression.

In addition to univariate analyses, we explored recurrence using two restricted multivariable models, each limited to two covariates to reduce overfitting. In these exploratory models, HPV DNA positivity and p16 loss/reduced expression remained associated with recurrence after a limited adjustment for disease extent (advanced Krouse stage). However, these findings should be interpreted cautiously and require prospective validation.

From a clinical standpoint, these findings reinforce that the postoperative risk stratification in IP should remain grounded in tumor extent (Krouse stage), anatomical origin, and smoking. While biomarkers such as p16 may offer additional information regarding recurrence risk, the data is not strong enough to warrant impacting surveillance intensity (frequency of endoscopy and imaging).

## 5. Limitations

Several limitations should be acknowledged. First, due to its retrospective design, no formal a priori power calculation was done. The sample size was determined by the number of eligible cases with sufficient available data, resulting in the exclusion of a substantial proportion of initially identified cases. Follow-up schedules and imaging indications were not standardized. There were only nine patients with malignant transformations, limiting statistical power for malignant predictors. HPV-related findings also require cautious interpretation. HPV testing was performed retrospectively on archived material and assessed HPV DNA only; HPV transcriptional activity (e.g., *E6/E7* mRNA) was not evaluated. Moreover, p16 is not a validated surrogate marker of transcriptionally active HPV in sinonasal inverted papilloma, and the concordance between p16 and HPV activity cannot be inferred from our data. Given the modest sample size, the limited number of recurrent events, and the number of variables tested, there is a risk of false-positive associations due to multiple comparisons; therefore, the observed HPV–recurrence association should be regarded as hypothesis-generating and requires validation in larger prospective cohorts using standardized assays, ideally including measures of HPV transcriptional activity. Finally, molecular profiling beyond HPV (e.g., *EGFR* pathway alterations and *TP53/CDKN2A* changes) was unavailable; therefore, we cannot attest to the literature that suggests an inflammatory heterogeneous microenvironment with mixed *Th1/Th2/Th17* cytokine responses and epithelial neutrophilia exists as a defining hallmark. HPV18, *EGFR* exon 20 mutations, p53 alterations, and p16 loss have been linked to recurrence and developing SCC [30]. This report contributes to just one of these areas.

## 6. Conclusions

In this retrospective analysis of 73 IPs with sufficient data to review, there were 14 recurrences and nine malignant transformations (of which three were noted at initial surgery). Recurrence was associated with a loss or reduction in p16 expression and, as expected, with advanced Krouse stage or difficult sites to resect fully (such as the frontal or sphenoid sinus or near the infraorbital nerve). In restricted exploratory multivariable models, the direction of these associations was consistent. Recurrence was also associated with smoking, longer pre-diagnosis symptom duration, and HPV+ DNA. Malignant transformation was associated with smoking but not with HPV status. Overall, these findings support the multifactorial nature of recurrence and malignant progression in inverted papilloma and highlight the need for prospective validation in larger cohorts.

## Figures and Tables

**Table 1:** Univariate analysis of factors associated with recurrence and malignant transformation of IPs.

Variable	Recurrent (*n* = 14)	Non-Recurrent (*n* = 59)	*p* Value	Malignant (*n* = 9)	Non-Malignant (*n* = 64)	*p* Value
**Age, median (IQR)**	45 (38–55)	44 (34–55)	0.88	46 (40–58)	44 (34–55)	0.92
**Sex (male), n (%)**	11 (79%)	44 (75%)	1.00	7 (78%)	48 (75%)	1.00
**Symptom duration (months), median (IQR)**	30 (18–60)	12 (6–24)	0.003	24 (12–48)	18 (6–36)	0.56
**Smoking**	12 (86%)	30 (51%)	0.03	9 (100%)	33 (52%)	0.01
**Allergic rhinitis**	4 (29%)	18 (31%)	1.00	2 (22%)	20 (31%)	0.72
**Left nasal cavity**	7 (50%)	31 (53%)	1.00	5 (56%)	33 (52%)	1.00
**Krouse stage III–IV**	12 (86%)	20 (34%)	<0.001	6 (67%)	26 (41%)	0.18
**Combined approach**	3 (21%)	4 (7%)	0.12	2 (22%)	5 (8%)	0.20
**Frontal sinus origin**	3 (21%)	1 (2%)	0.02	1 (11%)	3 (5%)	0.39
**HPV DNA positive**	7 (50%)	13 (22%)	0.048	2 (22%)	18 (28%)	1.00

Notes: Continuous variables are presented as median (IQR) and compared using a Mann–Whitney U test. Categorical variables are presented as *n* (%) and compared using a Fisher’s exact test.

**Table 2:** Restricted exploratory multivariable logistic regression models for recurrence.

Variable	Model A Krouse + HPV aOR (95% CI)	*p*-Value	Model B Krouse + p16 aOR (95% CI)	*p*-Value
**Advanced Krouse stage (III–IV vs. I–II)**	5.84 (1.31–26.07)	0.021	6.10 (1.55–24.05)	0.010
**HPV DNA positivity** **(positive vs. negative)**	4.12 (1.02–16.61)	0.047	-	-
**p16 loss/reduced vs. retained**	-	-	4.35 (1.06–17.88)	0.041

Note: Each model was restricted to two covariates only to reduce overfitting, given the limited number of recurrent events.

**Table 3:** HPV DNA status, genotypes, and clinical outcomes in sinonasal inverted papilloma (*n* = 73).

Category	Subgroup	HPV+, *n*(%)	HPV−, *n*(%)	Total, *n*	*p*-Value *
**Outcome**	Overall cohort	20 (27%)	53 (73%)	73	-
	Recurrence	7 (50%)	7 (50%)	14	0.048
	No recurrence	13 (22%)	46 (78%)	59	-
	Malignant transformation	2 (22%)	7 (78%)	9	1.00
	No malignant transformation	18 (28%)	46 (72%)	64	-
**Genotype**	HPV11	7 (35%)		20	
	HPV16	5 (25%)		20	
	HPV18	2 (10%)		20	
	HPV6	2 (10%)		20	
	Co-infection (any)	3 (15%)		20	

Notes: Outcome percentages are calculated within each subgroup (row total). Genotype percentages are calculated using HPV-positive tumors (*n* = 20) as the denominator. * Fisher’s exact test (two-sided) for HPV DNA positivity vs. recurrence and vs. malignant transformation. Co-infections included HPV6/44 (*n* = 1), HPV11/42 (*n* = 1), and HPV16/18 (*n* = 1).

## Data Availability

The raw data supporting the conclusions of this article will be made available by the authors on request.

## Abbreviations

The following abbreviations are used in this manuscript:

Full term	Abbreviation
Sinonasal inverted papilloma	IP
Human papillomavirus	HPV
Squamous cell carcinoma	SCC
Immunohistochemistry	IHC
Formalin-fixed, paraffin-embedded	FFPE
